# Review of Laboratory Methods to Determine HDL and LDL Subclasses and Their Clinical Importance

**DOI:** 10.31083/j.rcm2304147

**Published:** 2022-04-18

**Authors:** Abdolreza Chary, Mehdi Hedayati

**Affiliations:** ^1^Prevention of Metabolic Disorders Research Center, Research Institute for Endocrine Science, Shaheed Beheshti University of Medical Sciences, 1985717413 Tehran, Iran; ^2^Cellular and Molecular Endocrine Research Center, Research Institute for Endocrine Sciences, Shahid Beheshti University of Medical Sciences, 1985717413 Tehran, Iran

**Keywords:** laboratory assessment, LDL, HDL, lipoprotein, review

## Abstract

Given the high prevalence of cardiovascular disease, accurate identification of methods for assessing lipoprotein subclasses, mainly low-density lipoprotein (LDL) and high-density lipoprotein (HDL) subfractions, can play an essential role in predicting the incidence of cardiovascular disease such as heart attack. LDL and HDL subclasses differ in size, surface charge, lipid and protein compositions, and biological role. There is no “gold standard” method for measuring the LDL and HDL subclasses or standardizing the different methods used to measure their subfractions. Over the past decades, various techniques have been introduced to evaluate and measure subclasses of these two lipoproteins, each with its own advantages and disadvantages. Development of laboratory methods that accurately HDL and LDL function must be developed and validated to high-throughput for clinical usage. In this review study, we tried to examine different methods of evaluating various subclasses of LDL and HDL by mentioning the strengths and weaknesses of each.

## 1. Introduction

There is a substantial correlation between the prevalence of Cardiovascular disease (CVD) and serum concentration of LDL [1, 2]. The results of numerous reported studies have shown that more than 50% of patients with CVD have an abnormality in their lipid profile [3, 4, 5]. Also, it has been reported that there was a contra association between serum levels of HDL and CVD incidence.

**Fig. 1.**
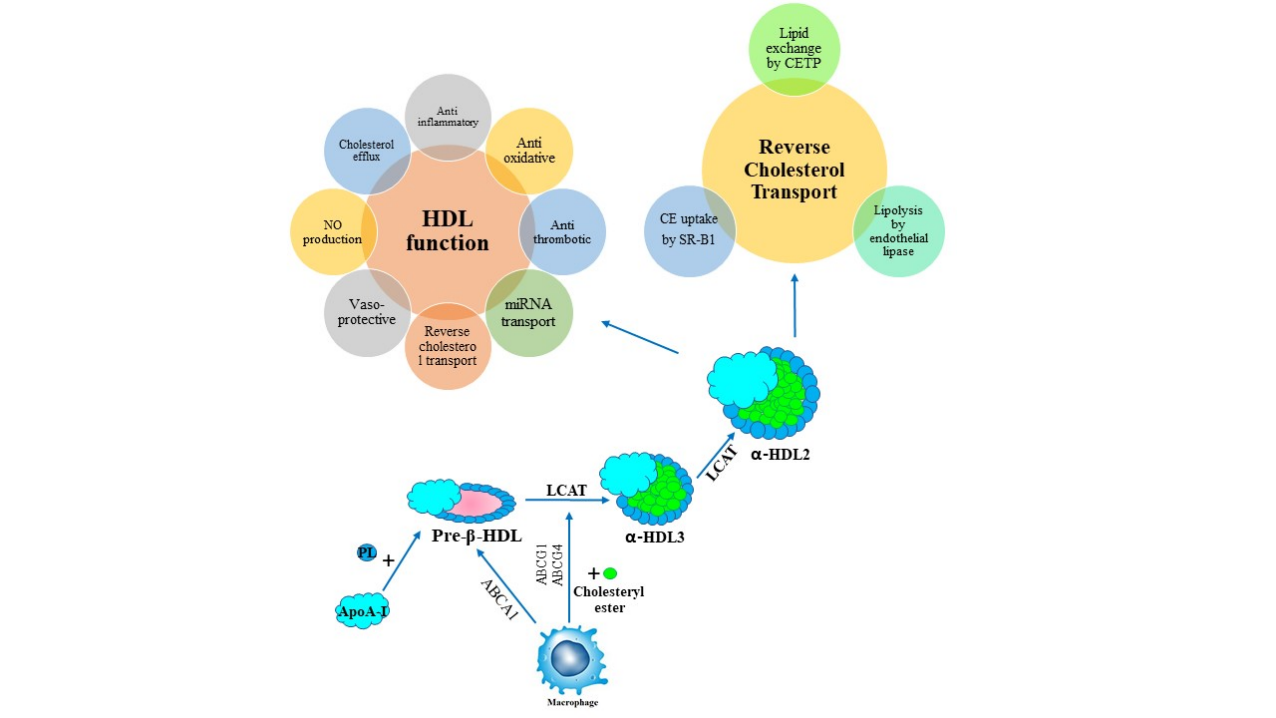
**High-density lipoprotein metabolism and reverse cholesterol transport**. ABCA1, ATP-binding cassette transporter A1; apoA-I, apolipoprotein A-I; CE, cholesteryl ester; CETP, cholesteryl ester transfer protein; Chol, cholesterol; HDL, high-density lipoprotein cholesterol; LCAT, lecitin-cholesterol acyltransferase; LDL, low-density lipoprotein cholesterol; LDLR, LDL-receptor; RCT, reverse cholesterol transfer; SR-BI, scavenger receptor class B type I; TG, triglyceride; VLDL, very low-density lipoprotein cholesterol.

On the other hand, treatment with some drugs such as statins, fibrates, bile acid resins, and niacin reduces the concentration of LDL cholesterol and, consequently, lessens cardiovascular disease incidence. Also, several epidemiological studies and prospective randomized trials have repeatedly demonstrated a strong inverse correlation between the magnitude of HDL concentration and coronary heart disease (CHD) [3]. Each 10 mg/dL (0.26 mmol/L) rise in HDL-C in the Framingham Heart Study reduced CHD mortality by 19% in males and 28% in females [4]. A summary of the HDL cholesterol metabolism process is shown in Fig. 1. As shown in Table 1 (Ref. [5, 6, 7, 8, 9, 10]), after synthesis in the liver and intestine, apoA-I is induced pre-β-HDL production through cell interaction. Small α-HDLs (HDL3) are then produced by adding more phospholipids to these nascent particles through interaction with peripheral tissues and esterification by the enzyme lecithin-cholesterol acyl transferase (LCAT). When enough phospholipids are added from the peripheral tissues to the HDL3 particles, HDL2 is produced. These HDL2 particles can play protective roles in various tissues against chronic diseases, especially cardiovascular diseases, and on the other hand, can enter the HDL remodeling cycle. Phospholipid transfer protein (PLTP) and cholesteryl ester transfer protein (CETP) contribute to HDL remodeling. Spherical HDL can be remodeled by lipases resulting in the reduction in HDL size, the formation of lipid-poor HDL particles, and the release of lipid-free apoA-I, which can restart the lipidation cycle [11].

**Table 1.** **Techniques used to isolate and measure LDL-C subclasses**.

Techniques	Method strengths	Weaknesses of the method
Agarose gel electrophoresis [5]	Relative accuracy, ability to evaluate abnormal lipoproteins, ability to evaluate changed samples, ability to maintain the gel for the visual record	Dependence on the skill level of the operator, the relative automation of the method
Ultracentrifugation sequential [6]	Ability to assess the composition of lipoproteins	Tedious; large sample volume
LRC method [7]	Well standardized, relative accuracy in measuring subclasses of lipoproteins	Tedious; large sample volume
HPLC [8]	Ability to measure LDL subfractions based on particle size	Need to LDL isolation by ultracentrifugation before chromatography
LipoPrint [9]	Clinically available measurement technique, lesser extent charge	Access in a small number of medical laboratories
NMR [10]	High accuracy, high-performance speed	Access in a small number of medical laboratories, calibration, and validation issues

HPLC, high pressure liquid chromatography; NMR, nuclear magnetic resonance; LRC, lipid research clinics.

In recent years, most medical guidelines and health-related associations have used traditional lipid profile biomarkers such as total cholesterol (TC), LDL, HDL, triglycerides (TG) to report risk factors associated with cardiovascular disease [12, 13].

However, in recent years, the evaluation of LDL and HDL subgroups has been introduced as a valid indicator of chronic diseases, especially CVD. The utility of non-traditional markers in risk assessment is best tested by combining them with a model that includes conventional risk factors [14, 15, 16].

The HDL family consists of different subclasses with a highly heterogeneous group of lipoproteins in a density range of 1.063 to 1.21 g/mL. Based on published ultracentrifuge analysis results, HDL includes different subfractions such as HDL1, HDL2, HDL3, and VHDL [17]. Different subfractions of HDL have been shown to have other physical-chemical and biochemical properties [18]. Researchers have reported that among different subgroups of LDL, smaller types are more easily absorbed by macrophages than larger types and are more susceptible to oxidative modification [19, 20]. On the other hand, the results of several studies have shown that oxidized LDL (Ox-LDL) is one of the main risk factors for cardiovascular disease and induces atherogenesis by promoting an inflammatory environment and lipid deposition in the arterial wall [21, 22].

Over the last few decades, the analytical chemistry correlated to HDL and LDL subclass evaluation has undergone considerable development [23, 24]. There is an increasing imperative to determine other HDL and LDL-related subclasses and functions in the context of current advances and identify biomarkers that better anticipate cardiovascular risk and can be used to determine the clinical favors of novel HDL LDL-targeted therapies [2, 25]. This need poses an opportunity for all scientists to take the lead in developing and validating such biomarkers. This review study focuses on laboratory assessment of HDL and LDL subclasses determination methods, which can play an important role in evaluating CVD and other chronic diseases.

## 2. LDL Characteristics

It has been reported that the LDL family includes a wide range of subclass from the very TG-enriched VLDL (density <1.006 kg/L) to the high dense small lipoprotein with a density range between 1.063 to 1.21 kg/L. Based on the hydrated density, LDL particles are traditionally defined as fractions with a mass between 1.006 and 1.063 kg/L as determined by preparative ultracentrifugation [26]. The so-called “broad-cut” LDL subclasses are heterogeneous with many distinct lipoprotein fractions: one of the most LDL infractions is intermediate-density lipoprotein (IDL) which has a high content of chylomicrons and very-low-density lipoprotein (VLDL) remnants, and its density is a range between 1.006–1.019 kg/L, the second subgroup is the main LDL, with a hydrated density of 1.019–1.063 kg/L and finally, lipoprotein(a) [Lp(a)] which is an LDL-like particle and has a density range between 1.05–1.21 kg/L [21].

## 3. Methods for Determination of LDL-C

Given that elevated levels of LDL and some of its subclasses are among the important risk factors involved in predicting cardiovascular disease, in recent years, several methods for evaluating LDL subclasses have been evaluated. Some of them are mentioned below.

## 4. Ultracentrifugation

Classification of LDL subfractions by ultracentrifugation methods requires either equilibrium or rate approaches. In this method, preparative fractionations can be performed by presenting serum or plasma to ultracentrifugation at the density of native non-protein solute, approximately 1.006 kg/L, to float TG-rich VLDL and chylomicrons retrieved by tube slicing or by aspirating with a syringe or pipette [27, 28]. The 1.006 kg/L lowest portion, the infranate consisting of LDL, HDL, IDL, and Lp(a), can be changed to 1.063 kg/L by adding a salt specially KBr and re-insert into the ultracentrifuge to float LDL, the amount of cholesterol, which is considered as an indicator of LDL. Due to the fact that performing the steps mentioned in the ultracentrifuge method for the separation of LDL subclasses is time consuming and technically tedious, simpler precipitation replaced it. In some research work, researchers routinely use a combination of sedimentation and ultracentrifugation methods to evaluate LDL subclasses [29]. As mentioned, one of the main impediments of the ultracentrifugation method is that it is time consuming and tedious, however, it is important to note that this method is very useful for the separation of highly labile lipoproteins and they can be changed with high salt concentrations and centrifugal forces. Another disadvantage of this method, as shown in Table 1, is that it requires many tubes and other laboratory equipment. Its accuracy varies from laboratory to laboratory and depends on the accuracy of the operator. In addition, the fractions obtained by this method may be very heterogeneous [30].

## 5. Electrophoresis Versus Nuclear Magnetic Resonance (NMR)

Different studies have shown that the evaluation of a lipoproteins subclass, especially LDL, provides an accurate estimate of lipoprotein metabolism and the risk of cardiovascular disease. Another method of evaluating lipoprotein subclasses is to separate them based on particle size using electrophoresis. Heterogeneity was first identified in subclasses of LDL using ultracentrifuge [31] and subsequently developed using density gradient ultracentrifugation (DGUC) [32] and gradient gel electrophoresis (GGE) [33]. Using this method, the researchers showed that plasma insulin is present in different subclasses based on density and size in three major fractions designated LDL-I to -III peak density intervals: 1.022–1.032 kg/L for LDL-I, 1.032–1.038 kg/L for LDL-II, and 1.038–1.050 kg/L for LDL-III) and a relatively minor fraction, LDL-IV (1.050–1.063 kg/L) [34]. Also, using this technique, the distribution pattern of LDL subclasses in a plasma sample was predicted. Various factors affect the pattern of insulin phenotype, including LDL size cutoffs, subfraction distributions, or algorithms such as electrophoretic mobility values [35] as this pattern includes either mostly large, buoyant LDL-I and II (pattern A), sdLDL-III (pattern B; LDL-III), or an intermediate pattern of LDL-II and III (pattern I; 40–50% LDL-III). HDL and LDL subfractions are usually separated on gradient polyacrylamide gel electrophoresis.

In the gel electrophoresis method, LDL subclasses move to the opposite pole in an electric field, and special fat-staining is used to identify them. Although this method was initially more of a qualitative evaluation technique, it was converted to a highly efficient quantitative method by depositing electrophoresis strips with chemical compounds such as phosphutangestate. A more convenient alternative to the modified agarose gel for LDL subclass separation is the addition of a magnesium-like cation that reduces the migration of β and pre-β lipoproteins, and this creates another band among pre-β and α lipoproteins, indicated to be Lp(a) by immunofixation. This creates another band between the pre-β and α lipoproteins, which is shown by Lp(a) immunofixation. Unlike previous methods, NMR spectroscopy is automated and provides rapid, direct separation of different subclasses of LDL and HDL [36]. In this technique, the methyl moieties of the LDL and other lipoprotein subfractions based on the particle’s sizes resonate at slightly different frequencies. Thus, smaller particles of lipoproteins resonate at lower frequencies. Lipoproteins can thus be assessed by either decomposing the core lipid methyl signal into individual signals [37, 38] or using statistical data to predict lipid levels on the whole methyl envelope [39].

Several studies have compared these two techniques in evaluating LDL subgroups. Blake *et al*. [40] found a significant correlation between LDL particle sizes assessed by NMR and bench GE in healthy participants. Similar findings were found between NMR with bench GE-assessed LDL patterns in the other two studies [41, 42]. Witte *et al*. [43] found that in 324 males and females with and without type 1 diabetes, the mean difference (or mean bias by B-A LOA analysis) among evaluated LDL size on NMR and peak LDL size on GE was 53.8Å (with NMR yielding smaller measurements). In another study conducted on the 131 healthy participants, it has been reported that the accuracy of the NMR in classifying participants according to LDL subfractions pattern, especially sdLDL was higher than the GE. On the other hand, one of the disadvantages of electrophoresis compared to newer methods is that it is somewhat labor-intensive and technique-sensitive [44].

## 6. High-performance Liquid Chromatography Versus Gel Filtration (HPLC versus GF)

One of the alternative techniques for assessment of the LDL subfractions is HPLC. The HPLC techniques are highly reproducible, accurate, and ideal for studying a large series of samples. Limited studies have compared the two methods and their accuracy in evaluating LDL subclasses. Scheffer PG. in a comparative study evaluated the particle size of LDL obtained by HPLC and GE methods in patients with type 2 diabetes. The results of this study showed that, LDL particle determinations were highly correlated between the two methods (r = 0.88, *p *< 0.0001). The mean particle diameters measured by HPLC were in close agreement with peak particle diameter values obtained by GF [31]. Also, B–A LOA revealed that the mean difference between LDL size on HPLC and on GE was 2.5Å (with HPLC yielding larger sizes). The 95% LOA were –6 and +10Å [45].

## 7. High-density Lipoproteins (HDL) Measurement Methods

Atherosclerotic cardiovascular disease, particularly coronary artery disease (CAD), is closely associated with plasma concentrations of HDL. These observations have been reported in various cohort studies with high sample sizes [1]. It has also been shown that even in patients with low LDL-C levels, HDL-C is still a valid predictor of heart disease [46]. Different subclasses of HDL vary in size (from 7 to 17 nm) and shape (unfolded protein, discoidal and spherical) and finally lipidome or proteome [47, 48]. HDL calculation has been standardized, and existing precipitation methods reach a high degree of precision for clinical purposes. However, so far, a “gold standard” technique for evaluating HDL subclasses has not been introduced, and different techniques have their advantages and disadvantages. In the following, we will evaluate some of the measurement techniques of HDL subclasses [49].

Techniques used to isolate and measure HDL-C subclasses is shown in Table 2 (Ref. [50, 51, 52, 53, 54, 55, 56, 57, 58, 59]). Techniques such as ultracentrifugation (UTC) [60], electrophoresis [61], HPLC, precipitation-based methods [62], direct measuring methods, and NMR have been routinely used in recent years in research work to evaluate different subclasses of insulin. However, one of the standard methods for HDL subfraction evaluation is the cholesterol content in HDL particles after precipitation of apoB containing lipoproteins [50].

**Table 2.** **Techniques used to isolate and measure HDL-C subclasses**.

Techniques	Advantages	Limitations
UC [51]	The first widely used method to separate the large buoyant HDL2 and the smaller, less buoyant HDL3 in plasma	The high salt concentration and the extreme g-force have been shown to significantly alter the composition and physicochemical properties of HDL that might influence some of the functional properties of the separated HDL fractions [59]
Single-step UTC: VAP assay [52]	Fast and use from whole plasma just one single predefined, narrow density ranges	Some HDL individual subpopulations cannot be isolated
Precipitation [53]	Separation based on ApoB depleted serum, cost, adequate access to this method in small laboratories	Proteins and apoE fraction confounders in HDL supernatant
Density gradient UTC [54]	Separation based on particle density, the standard method for lipoprotein method	High ionic strength and centrifugal force, High salt content that may affect the accuracy of the results
Gradient gel ND-PAGGE [55]	Separation by particle size, a sensitive method for evaluating insulin subclasses	Unable to separate preβ-2 populations, low access to laboratories, little information to predict cardiovascular disease
HPLC [50]	Separation based on the HDL particle size, rapid and accurate method	Access only in specialized laboratories, Albumin coelution with HDL fractions
NMR spectroscopy [56]	Separation based on the NMR signal of purified HDL, one of the convenient methods for measuring high volume samples, no prior sample manipulation	Lack of information on the composition of HDL subclasses, Inability to measure HDL subclasses with equal accuracy, Lack of detection of preβ-1HDL subclass
LCAT assay [57]	Separation based on the Fractional esterification rate, less cost, faster performance	May not measure the initial esterification rate and may not reflect the turnover of cholesterol
LipoPrint [58]	Clinically available measurement technique, lesser extent charge	Access in a small number of medical laboratories

LCAT, lecithin cholesterol acyltransferase; ND-PAGGE, non-denaturant polyacrylamide gradient gel electrophoresis; HPLC, high-performance liquid chromatography; NMR, nuclear magnetic resonance; VAP, vertical auto profile; UC, density-gradient ultracentrifugation; UTC, ultracentrifugation.

## 8. Precipitation Methods for the Measurement of HDL Subfraction

In this method, HDL-C is first separated by precipitating apoB containing lipoproteins from the serum samples, and in this process, researchers were used from a combination of polyanions, especially heparin–MnCl2, dextran sulfate–MgCl2 or phosphotungstate–MgCl2 and a divalent cation, such as magnesium, heparin–manganese, or calcium [63]. One of the advantages of this method is that it does not depend much on the operator’s skill and it is somewhat easy and fast to do, and it can evaluate HDL subgroups in both serum and plasma samples [64]. One of the drawbacks of this method by some researchers is the incomplete deposition of apo-B [65]. Also, some environmental factors (such as pH, ionic strength, temperature, and cryopreservation) and disease conditions such as hypertriglyceridemia, inflammation maybe affect the accuracy of the results obtained by this method [66].

## 9. Density Gradient Fractionation of HDL

One of the oldest methods for evaluating HDL-C subclasses is analytical UTC with density gradient flotation. The UTC density gradient process is based on density gradients. This technique is layered on the surface of a NaCl-KBr gradient using a swinging-rotor, plasma, or serum, developed by the sequential layering of 4 separate densities of salt solution at +15 ^∘^C. One of the positive points of using this method is that it can simultaneously separate the subclasses of HDL and LDL [67, 68].

## 10. Vertical Auto Profile (VAP)

This technique can directly evaluate the four main lipoproteins as well as lipoprotein (a) [Lp(a)] [69, 70]. This method is an inverted rate zonal, single vertical spin, density gradient ultra-centrifugation technique that separates all lipoproteins in <1 h. One of the advantages of this method, as mentioned, is the high speed of lipoprotein separation due to the use of a short horizontal axis of the centrifuge tube. This method has been proposed as one of the sensitive techniques for isolating HDL subclasses, especially HDL2 and HDL3. Another advantage of this method is that it costs less than other methods. However, some studies have shown that this method has less correlation in the separation of lipoprotein subclasses than other techniques such as NMR and ND-PAGGE (non-denaturant polyacrylamide gradient gel electrophoresis) [71, 72].

## 11. Capillary Isotachophoresis (cITP)

In this technique, plasma lipoproteins are isolated based on their electrophoretic charge. In this method, lipoproteins are specifically stained with fluorescent lipophilic dye before separation [73]. Using this method, insulin lipoproteins are isolated in three major subclasses based on their electrophoretic motion pattern, including fast (f): only α-migrating HDL, intermediate (i): HDL particles rich in cholesterol, apoA-II, apoE and apoC, and slow migrating (s) HDL: consisted of both α and preβ-migrating HDL [73, 74]. This technique is used in some studies that evaluate the effects of HDL boosting or LDL -lowering drugs or in studies performed on patients with hypercholesterolemia [75, 76, 77]. Other advantages of this method are high sensitivity and the ability to perform with a small serum sample.

However, one of the main drawbacks of this method is the limited potential of quantification since the amount and fluorescence yield of the dye incorporated into lipoproteins is likely to vary with in-between lipoprotein subpopulations due to interindividual variations in their lipid content [78, 79].

## 12. NMR

This method is one of the very fast techniques for evaluating HDL subclasses. In this method, the separation of different subclasses of HDL through the emitted NMR and the amplitude of oscillation of this NMR, which is specific to each subgroup and can be measured. Also, in this method, using proton spectroscopy, the particle size of each subgroup is accurately measured. With the help of this method, HDL subgroups are classified into three small, medium, and large groups, with a size range of 7 to 14 nm [80].

## 13. What the Clinicians Need to Know

Although there are different methods for evaluating LDL and HDL subfractions and they have been evaluated in some studies [59, 81], most of these methods are expensive and in some areas, especially in developing countries and low-income countries are not available. Various studies have evaluated the value of using different subclasses of LDL and HDL in predicting different diseases. Zeljkovic *et al*. [82] in a cross-sectional study on 200 hospitalised patient with acute ischemic stroke (AIS) were evaluated the association between HDL and LDL subclasses with AIS prediction. The results of this study showed that AIS patients had significantly more LDL III and IVb, but less LDL I and II particles. They also had significantly smaller HDL size, more HDL 3a, 3b and 3c and less HDL 2b subclasses. In this study, LDL and HDL particles were separated by gradient gel electrophoresisand serum lipid parameters were measured by standard laboratory methods. In another study, Oravec *et al*. [83] were assessed the association between HDL subfractions, which analysed by electrophoresis method, in patients with CVD and they found that In the patient group with the diagnosis of arterial hypertension and coronary heart disease, the large HDL subclass was significantly decreased and the small HDL subclass was increased. A number of other studies have pointed to the protective role of some HDL subgroups such as HDL-3 against the CVD [84]. In addition to CVD, some studies have even evaluated the association of LDL and HDL subfractions with other chronic diseases. Stevanovic *et al*. [85] in a case-control study included 84 patients with newly diagnosed colorectal cancer and 92 controls were evaluated LDL and HDL subclasses by gradient gel electrophoresis and found that patients had significantly smaller LDL and HDL diameters and greater proportion of small, dense LDL particles than controls. They concluded that decreased LDL and HDL diameters were independent predictors of colorectal cancer .

Generally, according to the recommendations of some scientific panels, there is no strong evidence for the use of HDL and LDL subtractions for initial clinical assessment or on-treatment management decisions in patients with low or intermediate risk for CVD [86]. On the other hand, some clinical conditions, such as Cholesteryl ester transfer protein (CETP) or CETP inhibitors, have been associated with striking changes in lipoprotein profile and composition. It also indicates that HDL-C concentration alone may not be adequate to assess the effect of this lipoprotein category on cardiovascular risk, as evidenced by the use of CETP inhibitors or the absence of a rise in carotid intima-media thickness in carriers of the apo A-I mutation despite very low HDL-C levels, implying that HDL quality, such as HDL subtype distribution and/or subtype features, maybe more helpful than HDL concentration [11, 87].

However, it seems that in areas where laboratory facilities are not available to assess LDL and HDL subclasses, clinicians can still use HDL and LDL concentrations to initial assess chronic disease. If facilities are available, it is recommended to use newer more accurate methods.

## 14. Summary and Perspectives

Given the importance of LDL and HDL in the pathogenesis of various diseases, especially cardiovascular disease, in this review study, we tried to explain the latest laboratory techniques used to evaluate these two lipoproteins by mentioning their strengths and weaknesses. To date, there is no “gold standard” method for measuring LDL and HDL subclasses, and all methods used have their strengths and weaknesses. It is necessary to develop newer methods to accurately measure the subtypes of these two lipoproteins and accurately identify their roles.

## Abbreviations

CVD, Cardiovascular disorders; DGUC, density gradient ultracentrifugation; CHD, coronary heart disease; IDL, Intermediate-Density Lipoprotein; CAD, Coronary Heart Disease; HDL-C, HDL cholesterol; GGE, gradient gel electrophoresis; NMR, Nuclear Magnetic Resonance; HPLC, High-performance Liquid Chromatography; VLDL, very-low-density lipoprotein; sdLDL, subfractions pattern especially; PAGE, polyacrylamide gel electrophoresis; VAP, Vertical auto profile.

## References

[1] Boekholdt SM, Arsenault BJ, Hovingh GK, Mora S, Pedersen TR, Larosa JC (2013). Levels and changes of HDL cholesterol and apolipoprotein AI in relation to risk of cardiovascular events among statin-treated patients: a meta-analysis. *Circulation*.

[2] Hedayati M, Daneshpour MS (2005). Evaluation of HDL-C determination methods. *Iranian Journal of Endocrinology and Metabolism*.

[3] Harangi M, Szentpéteri A, Nádró B, Lőrincz H, Seres I, Páll D (2017). HDL subfraction distribution and HDL function in untreated dyslipidemic patients. *Vessel Plus*.

[4] Hamer M, O’Donovan G, Stamatakis E (2018). High-Density Lipoprotein Cholesterol and Mortality: too much of a Good Thing. *Arteriosclerosis, Thrombosis, and Vascular Biology*.

[5] Nauck M, Winkler K, März W, Wieland H (1995). Quantitative determination of high-, low-, and very-low-density lipoproteins and lipoprotein(a) by agarose gel electrophoresis and enzymatic cholesterol staining. *Clinical Chemistry*.

[6] Brousseau T, Clavey V, Bard JM, Fruchart JC (1993). Sequential ultracentrifugation micromethod for separation of serum lipoproteins and assays of lipids, apolipoproteins, and lipoprotein particles. *Clinical Chemistry*.

[7] Kerscher L, Schiefer S, Draeger B, Maier J, Ziegenhorn J (1985). Precipitation methods for the determination of LDL-cholesterol. *Clinical Biochemistry*.

[8] Cazzolato G, Avogaro P, Bittolo-Bon G (1991). Characterization of a more electronegatively charged LDL subfraction by ion exchange HPLC. *Free Radical Biology and Medicine*.

[9] Varady KA, Lamarche B (2011). Lipoprint adequately estimates LDL size distribution, but not absolute size, versus polyacrylamide gradient gel electrophoresis. *Lipids*.

[10] Matyus SP, Braun PJ, Wolak-Dinsmore J, Jeyarajah EJ, Shalaurova I, Xu Y (2014). NMR measurement of LDL particle number using the Vantera® Clinical Analyzer. *Clinical Biochemistry*.

[11] Pirillo A, Norata GD, Catapano AL (2013). High-density lipoprotein subfractions-what the clinicians need to know. *Cardiology*.

[12] Parish S, Offer A, Clarke R, Hopewell JC, Hill MR, Otvos JD (2012). Lipids and lipoproteins and risk of different vascular events in the MRC/BHF Heart Protection Study. *Circulation*.

[13] Perk J, De Backer G, Gohlke H, Graham I, Reiner Z, Verschuren M (2012). European Guidelines on cardiovascular disease prevention in clinical practice (version 2012) The Fifth Joint Task Force of the European Society of Cardiology and Other Societies on Cardiovascular Disease Prevention in Clinical Practice (constituted by representatives of nine societies and by invited experts) Developed with the special contribution of the European Association for Cardiovascular Prevention & Rehabilitation (EACPR). *European Heart Journal*.

[14] Hlatky MA, Greenland P, Arnett DK, Ballantyne CM, Criqui MH, Elkind MSV (2009). Criteria for evaluation of novel markers of cardiovascular risk: a scientific statement from the American Heart Association. *Circulation*.

[15] Moons KGM, Kengne AP, Woodward M, Royston P, Vergouwe Y, Altman DG (2012). Risk prediction models: I. Development, internal validation, and assessing the incremental value of a new (bio)marker. *Heart*.

[16] Brown WV (2020). Methods of Calculating Low-Density Lipoprotein Cholesterol Level. *JAMA Cardiology*.

[17] Kostner G, Laggner P, Fruchart J, Shepherd J (2019). Chemical and physical properties of lipoproteins. *Human Plasma Lipoproteins*.

[18] Kostner GM, Scharnagl H, Kostner K, Maerz W, Schwandt P, Parhofer KG (2006). Zusammensetzung und Stoffwechsel der Lipoproteine. *Handbuch der Fettstoffwechselstörungen*.

[19] Berneis KK, Krauss RM (2002). Metabolic origins and clinical significance of LDL heterogeneity. *Journal of Lipid Research*.

[20] Borén J, Chapman MJ, Krauss RM, Packard CJ, Bentzon JF, Binder CJ (2020). Low-density lipoproteins cause atherosclerotic cardiovascular disease: pathophysiological, genetic, and therapeutic insights: a consensus statement from the European Atherosclerosis Society Consensus Panel. *European Heart Journal*.

[21] Trpkovic A, Resanovic I, Stanimirovic J, Radak D, Mousa SA, Cenic-Milosevic D (2015). Oxidized low-density lipoprotein as a biomarker of cardiovascular diseases. *Critical Reviews in Clinical Laboratory Sciences*.

[22] Gao S, Liu J (2017). Association between circulating oxidized low-density lipoprotein and atherosclerotic cardiovascular disease. *Chronic Diseases and Translational Medicine*.

[23] Warnick GR, Nauck M, Rifai N (2001). Evolution of methods for measurement of HDL-cholesterol: from ultracentrifugation to homogeneous assays. *Clinical Chemistry*.

[24] Langlois MR, Blaton VH (2006). Historical milestones in measurement of HDL-cholesterol: impact on clinical and laboratory practice. *Clinica Chimica Acta*.

[25] Hosseinzadeh S, Pakizehkar S, Hedayati M (2020). High-Density Lipoprotein Measurement Methods: From Precipitation to Nuclear Magnetic Resonance (NMR). *Iranian Journal of Endocrinology and Metabolism*.

[26] Nauck M, Warnick GR, Rifai N (2002). Methods for Measurement of LDL-Cholesterol: a Critical Assessment of Direct Measurement by Homogeneous Assays versus Calculation. *Clinical Chemistry*.

[27] Shiffman D, Louie JZ, Caulfield MP, Nilsson PM, Devlin JJ, Melander O (2017). LDL subfractions are associated with incident cardiovascular disease in the Malmö Prevention Project Study. *Atherosclerosis*.

[28] Lindgren F, Jensen L, Hatch F (1972). The isolation and quantitative analysis of serum lipoproteins. *Blood Lipids and Lipoproteins: Quantitation, Composition and Metabolism*.

[29] Siri-Tarino PW, Krauss RM (2016). The early years of lipoprotein research: from discovery to clinical application. *Journal of Lipid Research*.

[30] Ivanova EA, Myasoedova VA, Melnichenko AA, Grechko AV, Orekhov AN (2017). Small Dense Low-Density Lipoprotein as Biomarker for Atherosclerotic Diseases. *Oxidative Medicine and Cellular Longevity*.

[31] Niimi M, Yan H, Chen Y, Wang Y, Fan J (2021). Isolation and analysis of plasma lipoproteins by ultracentrifugation. *Journal of Visualized Experiments*.

[32] Krauss RM, Burke DJ (1982). Identification of multiple subclasses of plasma low density lipoproteins in normal humans. *Journal of Lipid Research*.

[33] Griffin BA, Caslake MJ, Yip B, Tait GW, Packard CJ, Shepherd J (1990). Rapid isolation of low density lipoprotein (LDL) subfractions from plasma by density gradient ultracentrifugation. *Atherosclerosis*.

[34] Krauss RM, Blanche PJ (1992). Detection and quantitation of LDL subfractions. *Current Opinion in Lipidology*.

[35] Chung M, Lichtenstein AH, Ip S, Lau J, Balk EM (2009). Comparability of methods for LDL subfraction determination: a systematic review. *Atherosclerosis*.

[36] Otvos JD (2002). Measurement of lipoprotein subclass profiles by nuclear magnetic resonance spectroscopy. *Clinical Laboratory*.

[37] Jeyarajah EJ, Cromwell WC, Otvos JD (2006). Lipoprotein particle analysis by nuclear magnetic resonance spectroscopy. *Clinics in Laboratory Medicine*.

[38] Kaess B, Fischer M, Baessler A, Stark K, Huber F, Kremer W (2008). The lipoprotein subfraction profile: heritability and identification of quantitative trait loci. *Journal of Lipid Research*.

[39] Soininen P, Kangas AJ, Würtz P, Tukiainen T, Tynkkynen T, Laatikainen R (2009). High-throughput serum NMR metabonomics for cost-effective holistic studies on systemic metabolism. *The Analyst*.

[40] Blake GJ, Otvos JD, Rifai N, Ridker PM (2002). Low-Density Lipoprotein Particle Concentration and Size as Determined by Nuclear Magnetic Resonance Spectroscopy as Predictors of Cardiovascular Disease in Women. *Circulation*.

[41] Ensign W, Hill N, Heward CB (2006). Disparate LDL phenotypic classification among 4 different methods assessing LDL particle characteristics. *Clinical Chemistry*.

[42] Hoefner DM, Hodel SD, O’Brien JF, Branum EL, Sun D, Meissner I (2001). Development of a rapid, quantitative method for LDL subfractionation with use of the Quantimetrix Lipoprint LDL System. *Clinical Chemistry*.

[43] Witte DR, Taskinen MR, Perttunen-Nio H, Van Tol A, Livingstone S, Colhoun HM (2004). Study of agreement between LDL size as measured by nuclear magnetic resonance and gradient gel electrophoresis. *Journal of Lipid Research*.

[44] Davy BM, Davy KP (2006). Comparison of assessment techniques: plasma lipid and lipoproteins related to the metabolic syndrome. *Lipids in Health and Disease*.

[45] Kotani K, Sakane N, Gugliucci A (2021). Similarity and differences in small dense low-density lipoprotein assessment: two methods compared. *Archives of Medical Sciences Atherosclerotic Diseases*.

[46] Toth PP, Barter PJ, Rosenson RS, Boden WE, Chapman MJ, Cuchel M (2013). High-density lipoproteins: a consensus statement from the National Lipid Association. *Journal of Clinical Lipidology*.

[47] Freeman LA (2013). Native–native 2D gel electrophoresis for HDL subpopulation analysis. *Lipoproteins and Cardiovascular Disease*.

[48] Sorci-Thomas MG, Owen JS, Fulp B, Bhat S, Zhu X, Parks JS (2012). Nascent high density lipoproteins formed by ABCA1 resemble lipid rafts and are structurally organized by three apoA-i monomers. *Journal of Lipid Research*.

[49] Yang HS, Hur M, Kim H, Kim SJ, Shin S, Somma SD (2020). HDL Subclass Analysis in Predicting Metabolic Syndrome in Koreans with High HDL Cholesterol Levels. *Annals of Laboratory Medicine*.

[50] Collins LA, Mirza SP, Kissebah AH, Olivier M (2010). Integrated approach for the comprehensive characterization of lipoproteins from human plasma using FPLC and nano-HPLC-tandem mass spectrometry. *Physiological Genomics*.

[51] Gofman JW, Young W, Tandy R (1966). Ischemic heart disease, atherosclerosis, and longevity. *Circulation*.

[52] Kulkarni KR, Marcovina SM, Krauss RM, Garber DW, Glasscock AM, Segrest JP (1997). Quantification of HDL2 and HDL3 cholesterol by the Vertical Auto Profile-II (VAP-II) methodology. *Journal of Lipid Research*.

[53] Patsch W, Brown SA, Morrisett JD, Gotto AM, Patsch JR (1989). A dual-precipitation method evaluated for measurement of cholesterol in high-density lipoprotein subfractions HDL2 and HDL3 in human plasma. *Clinical Chemistry*.

[54] Silverman DI, Ginsburg GS, Pasternak RC (1993). High-density lipoprotein subfractions. *The American Journal of Medicine*.

[55] De Lalla O, Gofman JW (1953). Ultracentrifugal analysis of serum lipoproteins.

[56] Ala-Korpela M, Lankinen N, Salminen A, Suna T, Soininen P, Laatikainen R (2007). The inherent accuracy of 1H NMR spectroscopy to quantify plasma lipoproteins is subclass dependent. *Atherosclerosis*.

[57] Albers JJ, Chen CH, Adolphson JL (1981). Lecithin:cholesterol acyltransferase (LCAT) mass; its relationship to LCAT activity and cholesterol esterification rate. *Journal of Lipid Research*.

[58] Li J-J, Zhang Y, Li S, Cui CJ, Zhu CG, Guo YL (2016). Large HDL subfraction but not HDL-C is closely linked with risk factors, coronary severity and outcomes in a cohort of nontreated patients with stable coronary artery disease: a prospective observational study. *Medicine*.

[59] Yang DD, Konigshofer Y, Dike J, Chang E, Mueller O (2010). Performance characteristics of a microfluidic Lab-on-Chip electrophoresis system for high-density lipoprotein (HDL) subfraction separation and measurement. *Clinical Chemistry and Laboratory Medicine*.

[60] Konishi T, Fujiwara R, Saito T, Satou N, Crofts N, Iwasaki I (2021). Human lipoproteins comprise at least 12 different classes that are lognormally distributed. *medRxiv*.

[61] Meilhac O, Tanaka S, Couret D (2020). High-density lipoproteins are bug scavengers. *Biomolecules*.

[62] Asztalos BF, Collins D, Horvath KV, Bloomfield HE, Robins SJ, Schaefer EJ (2008). Relation of gemfibrozil treatment and high-density lipoprotein subpopulation profile with cardiovascular events in the Veterans Affairs High-Density Lipoprotein Intervention Trial. *Metabolism*.

[63] Mulinge M (2018). Comparison of Direct and Precipitation Methods for Estimation of Major Serum Lipoproteins in Hypertensive Patients. http://ir.jkuat.ac.ke/handle/123456789/4585.

[64] Hirano T, Nohtomi K, Koba S, Muroi A, Ito Y (2008). A simple and precise method for measuring HDL-cholesterol subfractions by a single precipitation followed by homogenous HDL-cholesterol assay. *Journal of Lipid Research*.

[65] Contois JH, Albert AL, Nguyen R (2014). Immunoprecipitation of apolipoprotein B-containing lipoproteins for isolation of HDL particles. *Clinica Chimica Acta*.

[66] Bhattacharjee R (2021). Laboratory Assessment of Lipoproteins: Principles and Pitfalls. *Manual of Lipidology*.

[67] Kontush A, Chantepie S, Chapman MJ (2003). Small, dense HDL particles exert potent protection of atherogenic LDL against oxidative stress. *Arteriosclerosis, Thrombosis, and Vascular Biology*.

[68] Guerin M, Lassel TS, Le Goff W, Farnier M, Chapman MJ (2000). Action of atorvastatin in combined hyperlipidemia: preferential reduction of cholesteryl ester transfer from HDL to VLDL1 particles. *Arteriosclerosis, Thrombosis, and Vascular Biology*.

[69] Chung BH, Wilkinson T, Geer JC, Segrest JP (1980). Preparative and quantitative isolation of plasma lipoproteins: rapid, single discontinuous density gradient ultracentrifugation in a vertical rotor. *Journal of Lipid Research*.

[70] Kulkarni KR (2006). Cholesterol profile measurement by vertical auto profile method. *Clinics in Laboratory Medicine*.

[71] May HT, Nelson JR, Kulkarni KR, Anderson JL, Horne BD, Bair TL (2013). A new ratio for better predicting future death/myocardial infarction than standard lipid measurements in women >50 years undergoing coronary angiography: the apolipoprotein A1 remnant ratio (Apo A1/[VLDL3+IDL]). *Lipids in Health and Disease*.

[72] Movva R, Rader DJ (2008). Laboratory Assessment of HDL Heterogeneity and Function. *Clinical Chemistry*.

[73] Schmitz G, Möllers C, Richter V (1997). Analytical capillary isotachophoresis of human serum lipoproteins. *Electrophoresis*.

[74] Böttcher A, Schlosser J, Kronenberg F, Dieplinger H, Knipping G, Lackner KJ (2000). Preparative free-solution isotachophoresis for separation of human plasma lipoproteins: apolipoprotein and lipid composition of HDL subfractions. *Journal of Lipid Research*.

[75] Shimizu T, Miura S, Tanigawa H, Kuwano T, Zhang B, Uehara Y (2014). Rosuvastatin activates ATP-binding cassette transporter a1-dependent efflux ex vivo and promotes reverse cholesterol transport in macrophage cells in mice fed a high-fat diet. *Arteriosclerosis, Thrombosis, and Vascular Biology*.

[76] Zhang B, Miura S, Yanagi D, Noda K, Nishikawa H, Matsunaga A (2008). Reduction of charge-modified LDL by statin therapy in patients with CHD or CHD risk factors and elevated LDL-C levels: the SPECIAL Study. *Atherosclerosis*.

[77] Iwata A, Miura S, Zhang B, Imaizumi S, Uehara Y, Shiomi M (2011). Antiatherogenic effects of newly developed apolipoprotein a-i mimetic peptide/phospholipid complexes against aortic plaque burden in Watanabe-heritable hyperlipidemic rabbits. *Atherosclerosis*.

[78] Zhang B, Böttcher A, Imaizumi S, Noda K, Schmitz G, Saku K (2007). Relation between charge-based apolipoprotein B-containing lipoprotein subfractions and remnant-like particle cholesterol levels. *Atherosclerosis*.

[79] Hafiane A, Genest J (2015). High density lipoproteins: Measurement techniques and potential biomarkers of cardiovascular risk. *BBA Clinical*.

[80] Labine LM, Simpson MJ (2020). The use of nuclear magnetic resonance (NMR) and mass spectrometry (MS)–based metabolomics in environmental exposure assessment. *Current Opinion in Environmental Science & Health*.

[81] Guo ZG, Li C, Zhong JK, Tu Y, Xie D (2012). Laboratory investigation of dysfunctional HDL. *Chemistry and Physics of Lipids*.

[82] Zeljkovic A, Vekic J, Spasojevic-Kalimanovska V, Jelic-Ivanovic Z, Bogavac-Stanojevic N, Gulan B (2010). LDL and HDL subclasses in acute ischemic stroke: prediction of risk and short-term mortality. *Atherosclerosis*.

[83] Oravec S, Dostal E, Dukát A, Gavorník P, Kucera M, Gruber K (2011). HDL subfractions analysis: a new laboratory diagnostic assay for patients with cardiovascular diseases and dyslipoproteinemia. *Neuro Endocrinology Letters*.

[84] Albers JJ, Slee A, Fleg JL, O’Brien KD, Marcovina SM (2016). Relationship of baseline HDL subclasses, small dense LDL and LDL triglyceride to cardiovascular events in the AIM-HIGH clinical trial. *Atherosclerosis*.

[85] Stevanovic M, Vekic J, Bogavac-Stanojevic N, Janac J, Stjepanovic Z, Zeljkovic D (2018). Significance of LDL and HDL subclasses characterization in the assessment of risk for colorectal cancer development. *Biochemia Medica*.

[86] Davidson MH, Ballantyne CM, Jacobson TA, Bittner VA, Braun LT, Brown AS (2011). Clinical utility of inflammatory markers and advanced lipoprotein testing: advice from an expert panel of lipid specialists. *Journal of Clinical Lipidology*.

[87] Asztalos BF, Sloop CH, Wong L, Roheim PS (1993). Two-dimensional electrophoresis of plasma lipoproteins: recognition of new apo a-i-containing subpopulations. *Biochimica Et Biophysica Acta*.

